# Untreated Patients Dying With AIDS Have Loss of Neocortical Neurons and Glia Cells

**DOI:** 10.3389/fnins.2019.01398

**Published:** 2020-01-15

**Authors:** Sanne Simone Kaalund, Annette Johansen, Katrine Fabricius, Bente Pakkenberg

**Affiliations:** ^1^Research Laboratory for Stereology and Neuroscience, Copenhagen University Hospital, Bispebjerg and Frederiksberg, Copenhagen, Denmark; ^2^Gubra, Hørsholm, Denmark; ^3^Institute of Clinical Medicine, Faculty of Health and Medical Sciences, University of Copenhagen, Copenhagen, Denmark

**Keywords:** AIDS, cerebral cortex, optical disectors, quantitative neuroanatomy, stereology

## Abstract

Untreated human immunodeficiency virus (HIV) depletes its host CD4 cells, ultimately leading to acquired immunodeficiency syndrome (AIDS). In brain, the HIV confines itself to astrocytes and microglia, the resident brain macrophages, but does not infect oligodendrocytes and neurons. Nonetheless, cognitive symptoms associated with HIV and AIDS are attributed to loss of axons and white matter damage. We used design-based stereology to estimate the numbers of neocortical neurons and glial cells (astrocytes, oligodendrocytes, and microglia), in a series of 12 patients dying with AIDS before the era of retroviral treatments, and in 13 age-matched control brains. Relative to the control material, there was a 19% loss of neocortical neuron (*p* = 0.04) and a 29% reduction of oligodendrocytes (*p* = 0.003) in the patients with AIDS, whereas astrocyte and microglia numbers did not differ between patients and controls. Furthermore, we saw a 17% reduction in mean hemispheric volume in the AIDS group (*p* = 0.0015), which was driven by neocortical and white matter loss (*p* < 0.05), while the archicortex, subcortical gray matter, and ventricular volumes were within normal limits. Our results confirm previous reports of neuronal loss in AIDS. The new finding of oligodendrocyte loss supports the proposal that HIV in the brain provokes demyelination and axonal dysfunction and suggests that remyelination treatment strategies may be beneficial to patients suffering from HIV-associated neurocognitive deficits.

## Introduction

According to The Joint United Nations Programme on human immunodeficiency virus (HIV) and acquired immunodeficiency syndrome (AIDS) (UNAIDS, 2018), 37.9 million people globally are living with HIV. Despite public health campaigns and effective antiviral treatments, where were 1.7 million new HIV infections recorded in 2018, and a morbidity of 7,70,000 people from AIDS-related illnesses (UNAIDS, 2018). This reflects a halving of the incidence and morbidity since (UNAIDS, 2018), which doubtless reflects the enormous efforts expended in disease control and treatment. Even though forty percent of patients diagnosed with AIDS present neurological signs or symptoms during the course of their infection, the topic of AIDS infection in the central nervous system (CNS) is relatively neglected (Navia et al., 1986; Petito et al., 1986). Post-mortem studies conducted early in the epidemic found pathological CNS alterations in 80% of patients (Navia et al., 1986; Petito et al., 1986). Computed tomography and magnetic resonance imaging studies indicated a progressive cerebral atrophy in AIDS, which is linked to neuronal loss (Pedersen et al., 1991; Raininko et al., 1992; Oster et al., 1995; Korbo et al., 2002). While atrophy is not always attested by post mortem brain weight, morphometric analyses performed on autopsy material from AIDS patients indicate a 11% reduction in neocortical volume by 11 and 55% dilation of mean ventricular volume (Oster et al., 1995). Neuronal loss may be restricted to the neocortex, since neuronal numbers were preserved in hippocampus of AIDS patients (Korbo and West, 2000), despite atrophy of the neuronal soma (Sá et al., 2000).

The CNS is a major target of HIV infection (Conomy, 1989; Glass et al., 2001), yet neuronal loss in HIV-infected patients must be secondary to infection of microglia resident in the brain. Furthermore, free virus particles may penetrate the CNS by crossing the capillary endothelial cells that comprise the blood-brain and blood-cerebrospinal fluid barriers, or be carried into brain by infected lymphocytes or monocytes (de Almeida et al., 2006). Once a CNS infection is established, neuronal injury likely occurs by indirect mechanisms such as toxicity from virus proteins, macrophage factors, cytokines, and chemokines, or due to a loss of neurotrophic factors. While combined antiretroviral therapy (cART) can reduce plasma viral load to undetectable levels, it remains unclear if other HIV reservoirs persist. To address this questions, Lamers et al. (2016) measured HIV DNA in various autopsy tissues from a series of 20 HIV+/cART-treated patients with low or undetectable anti mortem viral loads and in plasma and cerebrospinal fluid (Lamers et al., 2016). Quantitative and droplet digital PCR identified the presence of HIV DNA in 48/87 brain tissues and 82/142 non-brain tissues at levels exceeding 200 HIV copies/million cell equivalents. Not one of the 20 cases was completely free of tissue HIV and abnormal histological findings, and all examined brain tissues demonstrated some degree of pathology, leading the authors to propose that HIV reservoirs was present in macrophage-rich tissues such as CNS and testis, despite complete clearance from plasma (Lamers et al., 2016).

A stereological study of the entire neocortex from AIDS patients dying prior to cART has previously shown a significant decrease in the total number of neocortical neurons (Oster et al., 1995). The individual extent of this neuron loss had no evident relationship with the presence of clinical dementia, HIV encephalitis or other opportunistic infections of the CNS Given that glial changes may contribute to the AIDS-related CNS pathology, we aimed in the present study to assess the effect of HIV-infection on the total number of glial cells in post-mortem neocortex from patients dying with AIDS. We undertook this study in a unique material of brains from patients dying before the advent of cART. Using design-based stereological methods, we estimated the glial sub-populations, i.e., astrocytes, oligodendrocytes and microglia, as well as neuron numbers, in the entire neocortex of the AIDS patients comparted to numbers in well-matched control material from patients without neurological disease.

## Materials and Methods

The initial material included brains from 50 patients who had died with AIDS, which had been collected consecutively from 1986 to 1989 at Hvidovre University Hospital, Copenhagen, in accordance with Danish laws on autopsied human tissue. An autopsy was carried out in each case, which included histological examination of tissues from the brain and internal organs. From among the 50 cases, we selected brains for detailed study based on a compilation of clinical and pathological data. Excluded were females, patients with intracranial space occupying lesions, and history of alcohol or other drug abuse. This selection left 12 brains from AIDS patients for further examination, of whom the six youngest patients were also included in the study by Fischer et al. (1999), and nine had also been part of a study by Oster et al. (1995). The duration of known HIV infection ranged from 5–42 months, with five patients having the HIV+ diagnosis for 6 months or less and the remaining seven patients having had the diagnosis since 2 or 3 years. Four of the AIDS patients had subjective and/or objective signs of dementia. The 12 AIDS patients (mean age 44.6 y; range 20–67 y) were group matched for age with the 13 control subjects (mean age 43.5 y; range 21–67 y). Table 1 shows the age, body height, post mortem interval, cause of death, and major autopsy and neuropathological findings in the subjects dying with AIDS. Also shown in Table 1 are the durations of HIV infection and AIDS diagnoses. The patient files did not indicate the precise extent of agonal weight loss, although all AIDS patients had lost weight to some degree and three were described as being emaciated. No gross brain abnormalities were seen at general autopsy. Control subjects had died from traffic accidents, homicide, or from cardiopulmonary diseases. Their body condition was otherwise normal, and there was no evidence of history of neurological or psychiatric disease. The clinical data for the control subjects are shown in Table 2. At autopsy, the brain stems were normal, including the pigmentation of substantia nigra. There were no tumors or neuronal glia inclusions, vasculitis or encephalitis.

**TABLE 1:** Clinical and demographic data for AIDS patients.

**#**	**Age**	**Body height (CM)**	**Brain weight (KG)**	**PMI (days)**	**Cause of death**	**Major autopsy findings**	**Neuropathological findings**	**HIV+ (months)**	**AIDS (months)**
1	20	170	1320	1	Kaposi sarcoma, PCP	Kaposi sarcoma	Edema, gliosis	30	13
2	26	173	1275	1	Pneumonia (atypical TB), polyneuropathy	Pneumonia, pneumo-peritonitis	Microglial nodular encephalitis	6	5
3	32	185	1465	2	PCP; emaciation, dementia	pneumonia	Meningoencephalitis (Cryptococcus)	26	19
4	37	183	1290	0.6	Kaposi sarcoma; PCP	Kaposi sarcoma, CMCV	CMV encephalitis	23	13
5	39	181	1280	1.3	Chronic herpes, oral candidiasis, emaciation, uremia	PCP	Microglial nodular encephalitis	5	1
6	40	187	1080	1	PCP, CMVP	PCP, CMVP	Edema, gliosis, microinfarction	36	1
7	46	176	1670	0.7	Pleural effusion, intestinal Kaposi sarcoma	Kaposi sarcoma in rectum	Edema, gliosis	5	5
8	51	170	1495	1	PCP, herpes zoster, CMV, retinitis	CMVP	Microglial nodular encephalitis	24	17
9	52	184	1435	1	Immunoblastic ML	Immunoblastic ML, PCP, CMVP	Edema, gliosis	6	
10	56	182	1450	3	Large cell ML, emaciation, dementia	Large cell ML, pneumonia	edema	37	1
11	57	170	1370	–	PCP, syphilis, CMV, cystitis, dementia	CMVP	CMV encephalitis	42	42
12	67	168	1340	0.6	Candida esophagitis, cryptococcosis, dementia	PCP	Edema, gliosis	6	4
Mean and	44.6	177	1373	1.2				20.5	11.0
range	20–67	168–187	1080–1670					5–42	1–42

**TABLE 2:** Clinical and demographic data for control.

**#**	**Age (years)**	**Body height (cm)**	**Brain weight (gram)**	**PMI (days)**	**Cause of death**
1	21	179	1600	2.5	Asthma
2	21	183	1485	4	Suicide
3	34	181	1230	0.3	Homicide
4	40	179	1620	2.5	Acute myocardial infarction
5	41	174	1432	1	Cardiomyopathy
6	43	170	1150	4	Acute myocardial infarction
7	43	176	1680	2	Acute myocardial infarction
8	43	175	1560	1	Acute myocardial infarction
9	44	185	1570	1	Pulmonary embolism
10	52	162	1365	2	Acute myocardial infarction
11	57	180	1393	1	Acute myocardial infarction
12	60	175	1500	1	Acute myocardial infarction
13	67	162	1330	1.5	Acute myocardial infarction
Mean and	43.5	176	1455	1.8	
range	21–67	162–185	1150–1620	0.3–4	

*PMI: Post-mortem interval.*

### Cell Characterization

**FIGURE 1:**
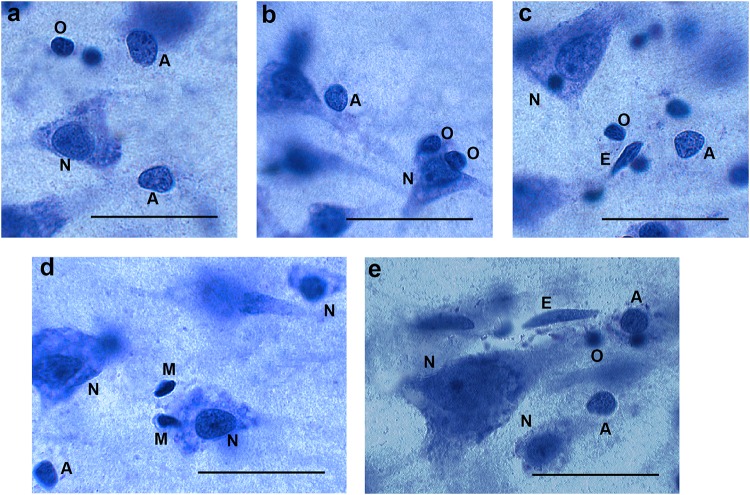
Different cell types in 40 μm thick sections (not all cells are in focus). Panels **(a−c)** from AIDS brains, panels **(d,e)** from control subjects. N = neurons, A = astroglia, O = oligodendroglia, M = microglia. E = endothelial cell (not counted). Bar = 30μm.

We relied on morphological identification of the different cell types, because immunohistochemical staining of specific glial markers (e.g., glial fibrillary acidic protein, S100, and CD11b) was not successful in all brains. The reliability of the identification of glial subtypes (astrocytes, oligodendrocytes, and microglia) was supported by successful immunohistochemical staining on selected cell types. We counted cells as neurons if they contained a single large nucleolus, in the nucleus had a typical, pale chromatin pattern within a triangular-shaped, rounded nucleus that was surrounded by a visible cytoplasm. Astrocytes were defined as cells with a round, pale nucleus, heterochromatin concentrated in granules in a rim below the nuclear membrane, and a relatively translucent cytoplasm. Astrocytes did not always have a small nucleolus; when present, it was most often located eccentrically. The nuclear membrane of astrocytes had a sharp profile and the cells were often seen as single, isolated cells (Pelvig et al., 2008; Karlsen and Pakkenberg, 2011). On the other hand, oligodendrocytes often occur in groups, and often in close proximity to neurons or blood vessels. Cells identified as oligodendrocytes had a small round or oval nucleus with dense chromatin, often surrounded by an artifact halo. Microglia cells are defined by their small elongated or comma-shaped nuclei containing dense peripheral chromatin (Pelvig et al., 2008; Karlsen and Pakkenberg, 2011). Examples are given in Figure 1. Using these criteria, we could assign an identification to more than 95% of cells, and therefore omitted the remainder from the analysis.

### Estimation of the Volume of the Neocortex and of Brain Cell Density

One cerebral hemisphere from each subject was used for the stereological study, and the other hemisphere underwent histopathological examination, using methods described in detail elsewhere (Regeur et al., 1994; Oster et al., 1995; Pakkenberg and Gundersen, 1997; Gredal et al., 2000). In brief, the hemisphere for stereology was embedded in 6% agar and cut into 7-mm-thick slabs. We estimated the surface area of neocortex by point counting; an average of 247 (range 167–316) points per neocortex were counted on the set of slabs, which resulted in a coefficient of error (CE = SEM/mean) of 3.5% for the cortical surface area. The hemispheric volume was calculated by multiplying the sum of neocortical areas for all slabs by the average slab thickness of all slabs. Starting at random, we sampled transcortical wedges uniformly from each neocortical region, the frontal-, temporal-, parietal and occipital lobe. Each wedge was cut into 2-mm-wide parallel bars and systematically randomly subsampled, such that each neocortical subregion was represented by about ten uniformly sampled bars. After embedding the bars in LKB-Historesin^®^ we cut one 35-μm-thick section from each bar, for staining with a modified Wolbach’s Giemsa stain. Neocortex was defined as the entire isocortex less the archicortex, which comprised the uncus, hippocampus, the parahippocampal gyrus, gyrus fornicatus, and the subcallosal area.

To estimate the total number of cells in each neocortical region *N*(cell, reg), we multiplied the regional neocortical reference volume, *V*(reg) by the regional numerical density, *N*_V_(cell/reg) as follows: *N* = *N*_V_(cell/reg) × *V*ref for *N*_V_ = ΣQ/ΣP × vol(dis). Here, ΣQ^–^ is the total number of cells counted in all disectors in a region and the term v(dis) is the total volume of these disectors, which equals the product of the area of the counting frame times the height of the disector, times the total number of disectors. For cell counting we used a modified BH-2 Olympus microscope equipped with an electronic microcator [Heidenhain(C)VRZ401] with digital readout for measuring movements in the *Z*-direction and a disector height of 15 μm. The area of counting frames were 102 μm^2^ in the three major lobes and 51 μm^2^ for the occipital lobe, and counting was performed with a ×60 oil immersion objective resulting in final on-screen magnifications of 2525×. The upper guard zone was set at 4 μm and the lower guard zone at 6 μm. The mean section thickness measured in every second disector was 26 μm (range 22–28 μm). Additionally, we confirmed the uniform distribution of neurons within the disector height by analyzing cell density throughout the *z*-distribution. All sections were coded during the process of stereological quantification. We estimated the extent of volumetric shrinkage, S_V_, for each neocortical region by comparing the volume of a 5 mm × 5 mm × 5 mm fixed but pre-embedded tissue to the estimated volume of the embedded and stained tissue sections of the same block after histological processing.

### Statistical Analysis

Results in the two groups were compared with an unpaired Student’s *t*-test, with the level for significance set at 0.05. If the normality test (Shapiro–Wilk) for a data set failed, we applied the non-parametric Mann–Whitney *U* test. We used the Pearson’s correlation coefficient to test for correlation between total numbers of neurons, oligodendrocytes, astrocytes or microglia, and disease duration.

For all data, the coefficient of variation (CV) equals the ratio SD/mean, which we report in parentheses throughout. In evaluating the precision of the counting estimates, the coefficient of error (CE = SEM/mean) provides the information necessary for determining whether the sampling is sufficiently precise at the various levels of the sampling scheme. There were no significant subject group differences between the CEs. The overall mean CE was 0.093 for the final estimates of the total number of neurons. Upon dividing the glial cells into subgroups, we obtained a CE of 0.078 for astrocytes, 0.068 for oligodendrocytes, and 0.26 for microglia, versus 0.056 for the complete set of neocortical glia cells.

## Results

### Patients With AIDS vs. Controls, Cell Numbers

**FIGURE 2:**
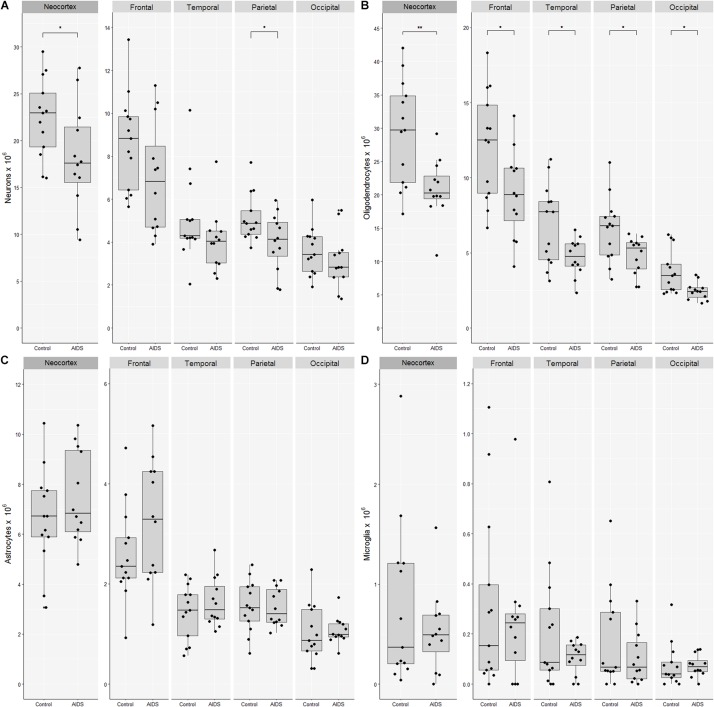
Loss of neocortical neurons and oligodendrocytes in AIDS patients. Combined boxplots and strip-charts showing the stereological cell estimates for **(A)** neurons, **(B)** oligodendrocytes, **(C)** astrocytes, and **(D)** microglia in controls and AIDS patients, for entire neocortex, and frontal, temporal, parietal, and occipital cortices. The boxes show the mean and the 25–75th percentile range, and whiskers the range of data within 1.5 of the interquartile range. Each point represents an estimate from an individual brain. ^∗^*p* < 0.05, ^∗∗^*p* < 0.01.

The mean number of neocortical neurons in control brains was 22.4 billion (CV = 0.18) compared with 18.2 billion (CV = 0.31) in AIDS patients. The apparent loss of 4.3 billion neurons (19%; *t*-test, *p* = 0.041) was significant for the neocortex as a whole and in the parietal lobe (*t*-test, *p* = 0.03), but not for the frontal (*t*-test, *p* = 0.09), the temporal (Mann–Whitney, *p* = 0.12), or occipital lobes (*t*-test, *p* = 0.33) (Figure 2). The mean number of neocortical oligodendrocytes was 29.4 billion (CV = 0.27) in control brains compared with 20.9 billion (CV = 0.21) in AIDS brains, corresponding to a loss of 8.5 billion (29%; *t*-test, *p* = 0.003) oligodendrocytes in the neocortex as a whole, which was likewise significant in each of the four lobes The mean number of neocortical astrocytes was 6.6 billion (CV = 0.30) in control subjects and 7.5 (CV = 0.25) in AIDS brains. The difference was not statistically significant for the entire neocortex (*t*-test, *p* = 0.30) nor for any of the four lobes alone (*t*-test, *p* > 0.05). The mean number of neocortical microglia was 0.77 billion (CV = 1.1) in control brains compared with 0.53 billion (CV = 0.78) in AIDS brains. The difference was not statistically significant in any lobe (*t*-test, *p* = 0.36).

The mean glia:neuron ratio was 1.6 in control and 1.6 in AIDS neocortices, which is not significantly different (*t*-test, *p* = 0.74). The sum of all glia and neurons in the entire neocortex was 59.2 × 10^9^ in controls and 47.1 × 10^9^ in AIDS, with the difference of 12.1 billion cells corresponding to 20% fewer neurons and glia in patients than in controls (*t*-test, *p* = 0.01).

### Cell Numbers and Disease Related Measures

There was no significant correlation between months of known HIV infection and number of neocortical neurons (Pearson’s correlation, *r* = −0.27, *p* = 0.40) or oligodendrocytes (Pearson’s correlation, *r* = −0.45, *p* = 0.12). There were no significant differences between the number of neurons or oligodendrocytes [*t*-test, *p*(neurons) = 0.88, *p*(oligo) = 0.48] in the four AIDS patients with signs of dementia, when compared with the eight AIDS patients without signs of dementia. The numbers of neurons and oligodendrocytes did not significantly correlate in the group of patients with AIDS (Pearson’s correlation, *r* = 0.46, *p* = 0.13), whereas that correlation reached significance in the control group (Pearson’s correlation, *r* = 0.55, *p* = 0.050). The numbers of cells (neither neurons nor glia) did not significantly correlate with age or height in patients (Pearson correlation, age *r* = 0.45, *p* = 0.14, height *r* = 0.12, *p* = 0.71) or controls (Pearson’s correlation, age *r* = 0.12, *p* = 0.71, height *r* = −0.03, *p* = 0.93).

### Patients With AIDS vs. Controls, Volumes, and Cortical Thickness

The brains from AIDS patients showed several signs of atrophy, including a 17% reduction in bilateral hemisphere volume [AIDS = 945 cm^3^ (CV = 0.15), controls = 1145 cm^3^ (CV = 0.12); *t*-test, *p* = 0.0015]. Among the four great lobes, the mean volumes of the frontal- and parietal cortices were significantly reduced in the patient group (Table 3).

**TABLE 3:** Cell numbers and volume by neocortical area.

		**Control**		**AIDS**		
		**mean**	**CV**	**mean**	**CV**	***p* value**
Frontal	Neurons	8.7 × 10^6^	0.26	7.0 × 10^6^	0.37	0.09
	Oligodendrocytes	12.2 × 10^6^	0.28	8.9 × 10^6^	0.32	0.02^∗^
	Astrocytes	2.6 × 10^6^	0.37	3.2 × 10^6^	0.38	0.15
	Microglia	0.3 × 10^6^	1.15	0.2 × 10^6^	1.06	0.78
	Volume, cm^3^	229.0	0.08	198.0	0.23	0.04^∗^

Temporal	Neurons	5.1 × 10^6^	0.40	4.1 × 10^6^	0.35	0.12
	Oligodendrocytes	7.0 × 10^6^	0.37	4.7 × 10^6^	0.26	0.02^∗^
	Astrocytes	1.4 × 10^6^	0.38	1.6 × 10^6^	0.30	0.35
	Microglia	0.3 × 10^6^	1.01	0.1 × 10^6^	0.62	0.60
	Volume, cm^3^	121.0	0.31	108.0	0.17	0.07

Parietal	Neurons	5.1 × 10^6^	0.22	4.0 × 10^6^	0.34	0.03^∗^
	Oligodendrocytes	6.7 × 10^6^	0.32	4.8 × 10^6^	0.26	0.02^∗^
	Astrocytes	1.5 × 10^6^	0.33	1.5 × 10^6^	0.25	0.96
	Microglia	0.2 × 10^6^	1.01	0.1 × 10^6^	1.00	0.57
	Volume, cm^3^	119.0	0.10	96.2	0.21	0.002^∗∗^

Occipital	Neurons	3.6 × 10^6^	0.31	3.1 × 10^6^	0.41	0.33
	Oligodendrocytes	3.8 × 10^6^	0.38	2.6 × 10^6^	0.25	0.01^∗^
	Astrocytes	1.0 × 10^6^	0.55	1.1 × 10^6^	0.21	0.83
	Microglia	0.1 × 10^6^	1.17	0.1 × 10^6^	0.57	0.47
	Volume, cm^3^	55.9	0.28	45.2	0.27	0.09

Neocortex	Neurons	22.4 × 10^6^	0.19	18.2 × 10^6^	0.31	0.04^∗^
	Oligodendrocytes	29.6 × 10^6^	0.30	21.0 × 10^6^	0.24	0.003^∗∗^
	Astrocytes	6.6 × 10^6^	0.25	7.5 × 10^6^	0.22	0.27
	Microglia	0.9 × 10^6^	1.24	0.5 × 10^6^	0.77	0.81
	Volume, cm^3^	524.9	0.16	447.4	0.09	0.002^∗∗^

*^∗^*p* < 0.05 and ^∗∗^*p* < 0.01.*

The white matter volume was also reduced in patients [AIDS = 431 cm^3^ (CV = 0.15), controls = 513 cm^3^ (CV = 0.25); *t*-test, *p* = 0.024], while there were no significant differences in archicortex volume [AIDS = 37.3 cm^3^ (CV = 0.41), controls = 39.3 cm^3^ (CV = 0.22); *t*-test, *p* = 0.69], the volume of the central gray structures [AIDS = 43.8 cm^3^ (CV = 0.16), controls = 51.6 cm^3^ (CV = 0.24); *t*-test, *p* = 0.063], mean ventricular volume [AIDS = 21.5 cm^3^ (CV = 0.37), controls = 16.0 cm^3^ (CV = 0.42); *t*-test, *p* = 0.071], or cortical thickness [AIDS = 2.53 mm (CV = 0.20), controls = 2.79 mm (CV = 0.36); *t*-test, *p* = 0.11].

No significant difference was found between the volumetric shrinkage in the groups of AIDS brains compared to controls (*t*-test, *p* = 0.50).

## Discussion

The major findings of this study are that numbers of astrocytes and microglia were normal, but the numbers of neurons and oligodendrocytes significantly were lower in brains from patients dying with AIDS in the time before cART treatment. The finding of a 19% reduction in the total number of neocortical neurons was not unexpected, since similar attrition has been reported in previous studies using unbiased stereological methods (19–27%) (Ketzler et al., 1990; Oster et al., 1995) and semi-quantitative stereological methods (Everall et al., 1991, 1993). The specific loss of the oligodendrocyte subpopulation of all glial cells may, in part, be explained by the generally close relationship between the numbers of oligodendroglia and neocortical neurons, which has been observed previously (Pelvig et al., 2008; Salvesen et al., 2017). Oligodendrocytes are specialized cells located in the gray matter and the subcortical white matter, which provide the myelin sheaths around axons enabling fast saltatory conduction of neuronal action potentials. Although there is some limited neurogenesis in the adult brain, oligodendrocyte precursor cells continue to divide, proliferate, and differentiate abundantly throughout life to secure a continuous turn-over of myelination (McLaurin and Yong, 1995). A reduction in oligodendrocyte numbers may therefore be interpreted to indicate increased cell death or some impairment in the proliferation, maturation, or differentiation process of oligodendrocyte precursor cells. There is growing evidence that HIV viral proteins are directly damaging to oligodendrocytes (Liu et al., 2016), and that widespread demyelination is characteristic of HIV-associated neurocognitive disorders (Jensen et al., 2019). Because oligodendrocytes and neurons do not express the primary receptor (CD4) permissive for HIV-1 entry into cells, they are unlikely to host an HIV-1 infection (Bracq et al., 2018). However, viral proteins released from infected astrocytes and microglia may be taken up by oligodendrocytes and cause damage. The best studied of these cytotoxic viral proteins is the trans-activator of transcription (Tat), which has indeed been detected within oligodendrocytes. This trans-activator of gene transcription directly affects survival, differentiation, and myelination properties of oligodendrocytes (O’Donnell et al., 2006; Zou et al., 2015, 2019; Liu et al., 2017; Stern et al., 2018). Further, the presence of a demyelination marker (IgG antibodies against myelin oligodendrocyte glycoprotein) in plasma and cerebrospinal fluid has been associated with higher viral burden and HIV-1 associated neurocognitive disorder (Lackner et al., 2010). This suggests that perturbation of oligodendrocyte function, potentially leading to net attrition, may be a primary cause of neuropathology patients with HIV.

Microglia constitute is an important class of CNS glial cells that perform various immune-modulatory functions in their capacity as resident macrophages. Microglia are the major CNS cell type productively infected by HIV-1, and most likely are a major contributor to the neurotoxicity observed during chronic HIV-1 infection (González-Scarano and Martín-García, 2005). Cacci et al. (2008) have demonstrated that prolonged (72 h) *in vitro* exposure to the bacterial endotoxin lipopolysaccharide (LPS) induces differentiation of microglia from rat brain to a potentially neuroprotective phenotype. They further investigated whether LPS regulated the properties of embryonic and adult neural precursor cells differently with respect to the “acute” phenotype acquired following a single (24 h) LPS stimulation. Their results indicated that activated microglia released pro-inflammatory cytokines which had a detrimental effect on neuronal survival rate, whereas “chronic activation” of microglia induced development of a neuroprotective phenotype characterized by secretion of anti-inflammatory cytokines. They further concluded that the nature, duration and strength of the microglial response to insults are tightly regulated by inputs both from neural cells and from components of the immune system. Finally, according to Ponomarev (Ponomarev et al., 2011; Chen et al., 2017), depending on the type of stimulus, microglia can assume a pro-inflammatory/antigen-resenting activation state or an anti-inflammatory/tissue-repairing activation state. The importance of macrophages and microglia in HIV-1 infection is further emphasized in simian immunodeficiency virus (SIV)-infected rhesus macaques with depletion of CD4+ T cells, in which persistence of the infection is sustained by macrophages and microglial cells (Micci et al., 2014). Thus, HIV has seemingly evolved to persist within the CNS in microglia, maintain a level of viral replication that is refractory to immune reactions and antiretroviral therapies that, in most patients, suffice to effectively ablate viral load in the peripheral blood (Chen et al., 2017).

Astrocytes are neuroectodermal-derived cells that form important component of the blood–brain-barrier. They support the function and metabolism of neurons, regulate the ionic homeostasis in o the CNS, and modulate synaptic transmission by the uptake of neurotransmitters. Further, they participate in regulating immune responses in the brain. Astrocytes can support low level replication of HIV, thus contributing to persistence of the virus to persist in the CNS as a latent infection (Dong and Benveniste, 2001; Albright et al., 2003; Alexaki et al., 2008; Narasipura et al., 2012).

In our study none of the patients had been treated for their HIV infection, having died between 1986 and 1989, before introduction of efficient anti-retroviral treatments. All died within 1–42 months after developing AIDS. Taken together, the results indicate that in the early days of the AIDS epidemic this specific group of AIDS patients was unable to sustain a normal population of neurons and oligodendroglia. In contrast, despite persistent infection of microglia and possibly astrocytes, these cell populations did not suffer any significant attrition.

Cerebral atrophy is one of the many manifestations of AIDS. The loss of a significant number of neurons and oligodendrocytes in the neocortex may suffice to explain the observed atrophy in the group of patients, and may be a factor in the neurological and cognitive symptoms seen in many AIDS patients, despite effective cART treatment (Navia et al., 1986). Thompson et al. (2005) have presented 3D maps of the pattern of vulnerable cortical regions, where atrophy is linked with cognitive decline and immune system suppression (Thompson et al., 2005). Using high-resolution MRI brain scans, they created maps of differential cortical gray-matter thickness in groups of AIDS patients and healthy controls, revealing 15% thinning of primary sensory, motor, and premotor cortices. We found that the brains from our pre-cART AIDS patients showed atrophy marked by reduced bilateral hemisphere volume and reduced neocortical volume. In agreement with the Thompson study we found region-specific volume loss within the cerebral cortex, namely significant reductions of frontal and parietal cortical volumes, whereas volume in temporal and occipital cortices was preserved. Further, the white matter volume was significantly reduced, while there were no significant changes in archicortex volume, the volume of the central gray structures, ventricular volume or cortical thickness. The lack of significant enlargement of the ventricular volume and the non-significant reduction in the cortical thickness stands in disagreement with our previous study reporting larger ventricles and thinner cortex in brains of some of these AIDS patients (Oster et al., 1995). However, the biological variation in these parameters was rather high (CV = 0.20–0.42), which should be taken into account in evaluation of results.

Among the several limitations of this study, we note the histological criteria for differentiating the different cell types. A more reliable separation of neurons and glia might have been obtained through the use of sensitive and robust antibodies for specific cell type markers, but this proved impossible in the present material due to the long-term formalin fixation and the poor penetration of antisera in plastic embedded tissue sections. However, our morphological criteria for cell differentiation have proved to be consistent in a number of previous stereological studies (e.g., Pelvig et al., 2008; Fabricius et al., 2013), and agreed with immunohistochemically derived populations (Hou et al., 2012). Further, the specific immunological markers do not always stain all of the cells in the target population due to variable expression of the antigen (Korzhevskii et al., 2005; Lyck et al., 2008). Strict definitions for the identification of glial cells is strengthened by our inclusion of a comparable control group. In addition, we note that our estimates of the sum of neurons and all three glia cell types is invulnerable to any uncertainty in distinguishing the various cells types; this sum was substantially lower in the brains of the group of AIDS patients compared with control subjects. A further limitation arises from the present design in which cell counting in entire brain lobes may have missed focal changes in cell populations, for example in sensorimotor cortex, and the possibility of localized gliosis cannot be rejected. This could explain why pathological examination has described gliosis in several of the included AIDS brains, despite present findings of reduced total glia cell number. Finally, due to a low number of microglia in neocortex, we sampled a small number of these cells compared with neurons, oligodendrocytes, and astrocytes, resulting in higher CE values for microglia. However, the biological variance of microglial numbers was also high, with CVs between 0.78 and 1.1, so the *mean* microglia numbers presented are still a robust indication of the true numbers, despite the lower precision.

The major strength of the present study lies in our use of design-based stereology. Whereas most earlier post mortem studies applied two-dimensional morphometric and histometric methods, resulting in 2D estimates or estimates based on size distribution of 2D neuronal profiles, we used unbiased stereology, which results in accurate estimation of total numbers and volumes in 3D. Further, the brains from patients dying with AIDS before the advent of cART treatment allows us to obtain knowledge about the significant brain changes that occurred in this unique archive material.

## Conclusion

In conclusion, we find that patients dying with AIDS in the early days of the AIDS epidemic suffered from a significant loss of neocortical neurons and oligodendroglia, with preservation of astrocytes and microglia numbers, despite the likely infection of these cell populations. As HIV-associated neurocognitive disorders remains a significant concern in patients who are otherwise successfully treated with cART, there is a need for more detailed understanding of how HIV infection and cART influence the number and function of neocortical neurons and glial cells.

## Data Availability Statement

The datasets used are available from the corresponding author on reasonable request.

## Ethics Statement

The study was approved by the Danish Ethical Committee, jr. 01-068/98 (KF) and the tissue bank jr.# 2007-58-0015.

## Author Contributions

BP and KF designed the study. KF and AJ contributed to the sample preparation and data collection. SK contributed to the data collection and analysis. BP, AJ, and SK wrote the manuscript. All authors read and reviewed the final version of the manuscript.

## Conflict of Interest

The authors declare that the research was conducted in the absence of any commercial or financial relationships that could be construed as a potential conflict of interest.
